# Depression and anxiety among children and adolescents pre and post COVID-19: A comparative meta-analysis

**DOI:** 10.3389/fpsyt.2022.917552

**Published:** 2022-08-03

**Authors:** Sifan Wang, Lin Chen, Hailiang Ran, Yusan Che, Die Fang, Hao Sun, Junwei Peng, Xuemeng Liang, Yuanyuan Xiao

**Affiliations:** Department of Epidemiology and Health Statistics, School of Public Health, Kunming Medical University, Kunming, China

**Keywords:** depression, anxiety, children and adolescent, COVID-19, meta-analysis

## Abstract

**Background:**

Published studies in comparing pre and post the COVID-19 pandemic depression and anxiety levels among children and adolescents yielded incongruent results. Therefore, there is a necessity to perform a timely meta-analysis to synthesize existing evidence.

**Methods:**

A total of 10 digital databases (PubMed, Web of Science, PsycINFO, the Cochrane Library, Embase, Scopus, ScienceDirect, CNKI, WanFang, CQVIP) were fully searched for eligible studies published before November 6, 2021. Based on quality assessment results, relevant data were extracted for eligible studies of higher quality. We combined standardized mean difference (SMD) or prevalence ratio (RR) for anxiety and depression pre and post the COVID-19 pandemic by using random-effects models. Sensitivity analysis was further performed to evaluate heterogeneity of included studies.

**Results:**

14,508 articles were preliminarily identified, and after stepwise screening process, 8 articles were included eventually. The results showed that the SMD for post COVID-19 anxiety score measured by GAD-7 was 0.12 (95% CI: 0.08, 0.17), an significant increase compared with pre COVID-19 period; the SMDs and 95% CIs for post COVID-18 depression scores measured by PHQ-9, PHQ-8, and MFQ were 0.17 (95% CI: 0.10, 0.24), 0.23 (95% CI: 0.08, 0.38), and 0.11 (95% CI: 0.06, 0.17), respectively, also significantly increased compared with pre COVID-19 period. The RR for depression was 2.54 (95% CI: 2.48, 2.60) in post COVID-19 period when compared with pre-pandemic.

**Conclusions:**

Children and adolescents reported deteriorated anxiety and depression levels after the COVID-19 pandemic. More attention should be paid to this vulnerable group. Effective, expedient, and practical intervention measures which are compatible with COVID-19 prevention and control policies should be developed and implemented to maintain mental health wellbeing of the youths.

## Introduction

Corona Virus Disease 2019 (COVID-19), caused by SARS-CoV-2, is a sudden, rapidly evolving pandemic (1). Since the outbreak of COVID-19 in Wuhan, China, it has swept the world, triggered unprecedented prevention and control measures like home isolation, school closure, and social distancing. The lockdowns and various security measures adopted in the pandemic have not only affected the economy, but also significantly changed the way people live (2). For instance, it has been reported that eating habits, sleep duration and quality, daily living, social, leisure, and educational activities were all prominently influenced by COVID-19 pandemic (3, 4).

COVID-19 pandemic also imposes great influence on mental health of the public (5). Kang et al. found that people were prone to mental health problems such as negative emotions, anxiety, depression, and post-traumatic stress syndrome (PTSD) after COVID-19 outbreak (6). Among all reported mental health problems during the COVID-19 pandemic, anxiety and depression are the most common (7, 8). Data released by Statistics Canada showed that between April and May 2020, nearly 24% of people reported average or poor mental health, a threefold increase from previous years, and 41% experienced moderate or severe anxiety symptoms (9). Statistics collected in June 2020 suggested that 18.4% of people in the United States experienced moderate to severe depression (10). During April to June 2020, a survey implemented in Canada and the United States showed that the impact of the COVID-19 pandemic on anxiety and depression in the general population was clear: 31% of people met the criteria for anxiety diagnosis and 29% people met the criteria for major depression diagnosis (11). An Ecuadorian cross-sectional study reported that social isolation has exacerbated anxiety and depression in the general population due to the outbreak of the COVID-19 pandemic (12).

Compared with other age groups, children and adolescent populations are observed increased risk of a variety of mood problems, typically depression and anxiety, and mood changes are associated with personality weaknesses that can flare up in the event of environmental triggers (13). During the COVID-19 pandemic, various environmental stressors, such as stay-at-home isolation, fear of contracting the virus, and dramatic changes in living environment, are the main causes of compromised psychological wellbeing for children and adolescents (14–16). A systematic review showed that COVID-19 pandemic caused psychological problems such as fear, concern, worry, attention deficits and hyperactivity, loneliness in children and adolescents, leading to a further deterioration of mental health in this vulnerable population (17). Another meta-analysis by Racine et al. found that the prevalence rates of anxiety and depression were 20.5 and 25.2% during COVID-19 based on commonly used symptom-based instruments (such as GDA-7, PHQ-9, MFQ), significantly higher than 12.9 and 11.6% reported by large cohort studies prior to COVID-19 (18).

To better elucidate the influence of COVID-19 on depression and anxiety of children and adolescents, some scholars measured and compared pre and post COVID-19 depression and anxiety prevalence rates, with incongruent findings. For instance, Li et al. found that both anxiety and depressive symptoms were less severe in post-pandemic period; another Canadian study of 146 adolescents aged 12-13 showed reduced anxiety symptoms and increased depressive symptoms after the COVID-19 pandemic (19, 20). Therefore, there is a necessity to combine existing evidence for yielding a more convincing conclusion. However, after careful literature review, no pertinent meta-analysis on this important topic has been published so far. In this meta-analysis, we aim to perform a timely synthesis for pre and post COVID-19 depression and anxiety among children and adolescents, so as to provide evidence for effective intervention.

## Methods

This meta-analysis was implemented and organized according to the Preferred Reporting Items for Systematic Reviews and Meta-Analyses (PRISMA) guidelines (21). We focused on published quantitative studies reported changes in anxiety and depression among general children and adolescents before and after COVID-19.

### Search strategy

We systematically searched seven English electronic databases (PubMed, Web of Science, PsycINFO, Cochrane Library, Embase, Scopus, ScienceDirect) and three Chinese databases (CNKI, CQVIP, WanFang). We searched studies published before November 6, 2021, the date that we performed literature searching. In the searching process, there is no need to put restrictions on the publication year, as nearly all articles related to COVID-19 were published from 2020 onward. According to the theme of our study, the keywords to be searched were “anxiety,” “depression,” “COVID-19,” “children” and “adolescents.” Every single keyword was searched separately. In reviews or other meta-analysis papers containing this keyword, we checked whether there are words with the same meaning but different expressions, and added these words into the search formula to expand the search scope. The following search term was used: (“mental health” OR “mental illness^*^” OR “mental disorder^*^” OR “psychological illness^*^” OR “Psychiatric Disorder^*^” OR anxiety OR angst OR nervousness OR hypervigilance OR social anxiety OR “anxiety disorder^*^” OR anxious OR “overanxious disorder^*^” OR depress^*^ OR “depressive disorder^*^” OR “Depressive Symptom^*^” OR “Emotional Depression” OR “affective disorder^*^” OR “mood disorder^*^”) AND (adolescent^*^ OR teenager^*^ OR child^*^ OR student^*^ OR juvenile^*^ OR school^*^ OR teen^*^ OR young OR youth^*^ OR minors) AND (COVID-19 OR coronavirus OR SARS-CoV-2 OR cov-19 OR 2019-nCoV OR severe acute respiratory syndrome Coronavirus2). Two authors (SW and LC) independently searched all the 10 databases with the same search term to ensure relevant studies were not missed.

Two authors (YC and DF) screened the titles and abstracts of the database records, retrieved full-text for qualification assessment, and independently qualified full-text records. If there is a disagreement between the two researchers, a third party (senior researcher, YX) will participate in discussion and resolve the inconsistencies. Some articles reported results that from the same study, in this case, we selected the articles which reported outcome variables that best match the inclusion criteria of the current study. References of the included studies were also carefully screened for potentially eligible studies.

### Inclusion and exclusion criteria

Studies were deemed eligible and included into the meta-analysis if they: (1) Quantitatively assessed and compared anxiety or depression in children and adolescents pre and post COVID-19; (2) Were of longitudinal design or two-wave (pre and post COVID-19) cross-sectional design; (3) Written in English or Chinese; (4) Reported depression or anxiety prevalence rates/reported means and standard deviations for depression or anxiety scores. Studies were further excluded if: (1) Were not original studies, like reviews, case reports, correspondences, comments; (2) Study subjects were not children or adolescents; (3) Analytical results provided were not suitable for synthesis; (4) Study subjects were clinical populations (patients with COVID-19 or another disease); (5) Contain small sample size subjects (<100).

### Study quality assessment

The Newcastle-Ottawa Quality Assessment Scale (NOS) is specifically designed to evaluate the quality of longitudinal studies in systematic reviews. NOS is consisted of 8 entries in 3 sections: (i) Selection: a. Representativeness of the exposed cohort; b. Selection of the non-exposed cohort; c. Ascertainment of exposure; d. Demonstration that outcome of interest was not present at start of study. (ii) Comparability: e. Comparability of cohorts on the basis of the design or analysis. (iii) Outcome: f. Assessment of outcome; g. Was follow-up long enough for outcomes to occur; h. Adequacy of follow-up of cohorts (22). The NOS uses a rating system to evaluate the quality of the literature, with a full score of 9: a study can be awarded a score of 1 for each item within the selection and outcome sections, a maximum score of 2 can be given for comparability. Two researchers (HR and XL) independently evaluated quality of the articles based on the above criteria. If discrepancies exist between two researchers' evaluations, the original article will be re-examined by a third person (senior researcher, YX). Studies with rated scores ≥ 6 are deemed high-quality, a rated score between 4 and 5 indicates moderate quality, a rated score ≤ 3 reflects low quality (23). All the studies included into the present meta-analysis with rated NOS scores no <5. Details of the NOS scores for included studies can be seen in Supplementary Table S1.

### Data extraction

Two authors (JP and HS) independently extracted data from the included studies. A standard information extraction table was designed in advance, which mainly includes the following information: author, publication year, origin of study, study period, study population, mean age of the participants, sample size, measuring scales used, reported prevalence or mean scores of anxiety or depression in pre and post COVID-19 periods. The information extracted by the two authors will be compared, if any disagreement exists, a senior researcher (YX) will perform data extraction again.

### Statistical analysis

Results were analyzed separately according to different outcome variables. For continuous outcome, measured by means and standard deviations of depression or anxiety scores, standardized mean difference (SMD) together with its 95% confidence interval (CI) was used; whereas for categorical outcome, measured by prevalence of depression or anxiety before and after COVID-19, relative risk (RR) with its 95% CI was used. Considering the included studies may use different depression or anxiety instruments, only studies used the same scale and reported the same outcome variable were combined. Therefore, the results were organized in subgroups.

Heterogeneity was tested by using the *I*^2^ statistic, with a *I*^2^> 50% considered substantial heterogeneity (24). If significant heterogeneity emerged, random-effects model will be used as recommended (25). A sensitivity analysis was further performed to evaluate the influence of each included individual study. All analyses were performed in STATA statistical software (Version 14, Stata Corp., College Station, Texas, U.S.) and R software (Version 4.2.0). Two-sided statistical tests were used consistently, with a *p* < 0.05 considered statistically significant.

## Results

**Figure 1:**
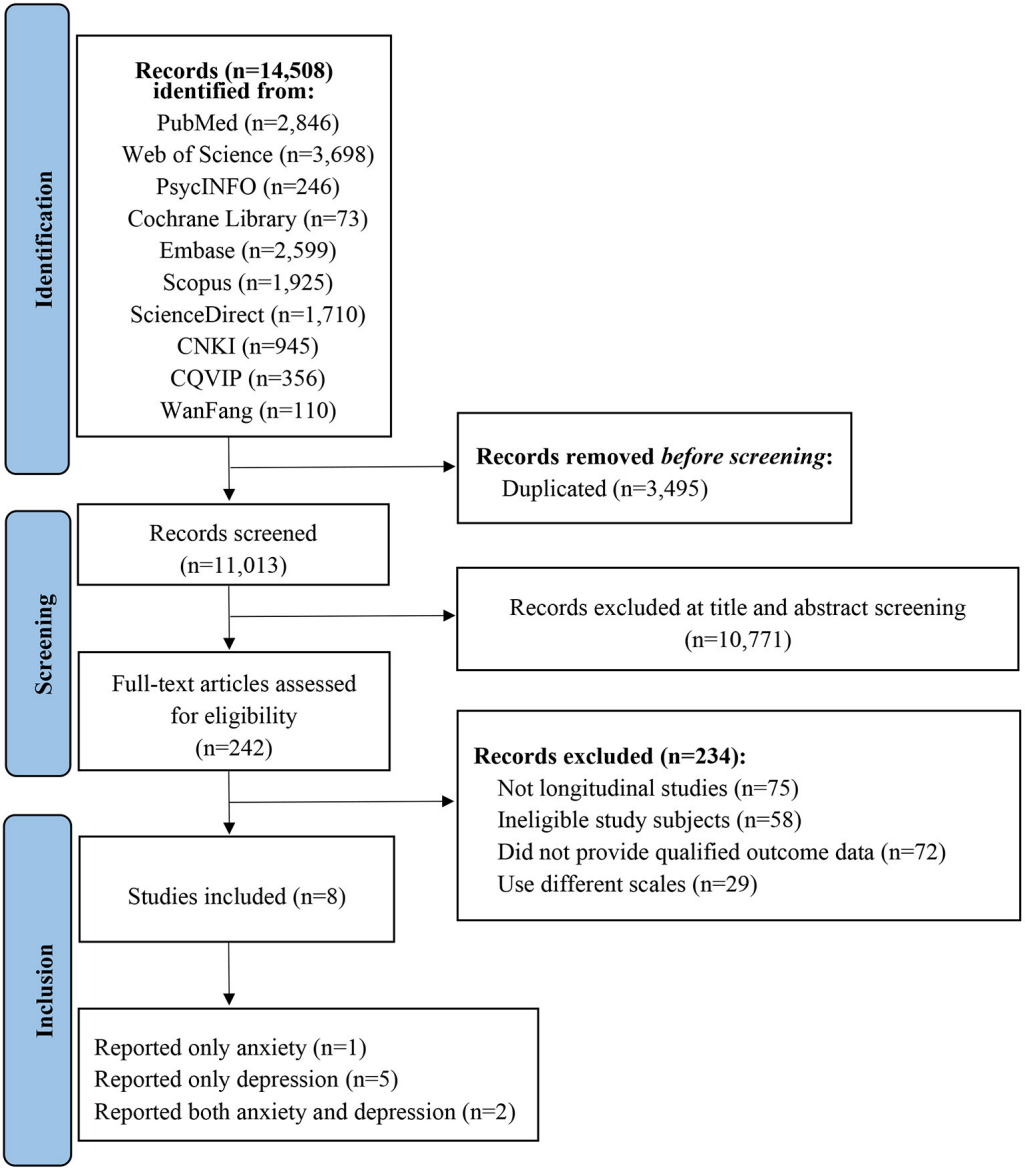
Flowchart of literature search and study selection according to the PRISMA standard.

The process of literature screening is shown in PRISMA flowchart (Figure 1). A total of 14,508 records were obtained initially from the 10 databases. The EndNote X9 software was used to remove duplicated articles, and 11,013 records remained. Two researchers (HR and YC) separately screened 11,013 articles for titles and abstracts and excluded 10,771 records. 242 articles were screened for full-text reading, and 8 articles were finally included in the study based on inclusion and exclusion criteria (26–33): 2 articles reported both anxiety and depression, 5 articles reported only depression, 1 article reported only anxiety. Therefore, 7 studies were included in meta-analysis of depression and 3 studies were included in meta-analysis of anxiety.

### Characteristics of included studies

Characteristics of the included studies are displayed in Table 1. Included studies were conducted in different countries: 5 in North America (4 in the US and 1 in Canada), 2 in Europe (1 in Greece and 1 in Norway), and 1 in Oceania (Australia). Sample sizes ranged from 100 (Jolliff 2021, the US) to 47,684 (Mayne 2021, the US). All studies used commonly used scales to measure anxiety and depression. The scales used to measure depression included: PHQ-9, PHQ-8, and MFQ. The GAD-7 was used to measure anxiety. All the instruments used by included studies had been repeatedly validated in children and adolescent populations (34–41). Among the 8 included studies, 6 reported scores (means with standard deviations) of depression or anxiety, 1 reported prevalence rates of depression, 1 reported both prevalence rates and scores of depression before and after the COVID-19 pandemic.

**Table 1:** Characteristics of the 8 studies included into the meta-analysis.

**References**	**Country**	**Study design**	**Sample size**		**Instrument**		**Age (years)**	**Anxiety, Mean (SD)**		**Depression** ***n*** **(%)/Mean (SD)**	
			**Pre^a^**	**Post^b^**	**Anxiety**	**Depression**		**Pre^a^**	**Post^b^**	**Pre^a^**	**Post^b^**
Giannopoulou et al. (27)	Greece	Cross-sectional	442	442	GAD-7	PHQ-9	SHSS^c^	7.93 ± 4.60	10.59 ± 5.79	212 (47.96) 10.82 ± 6.24	278 (62.89) 13.59 ± 7.25
Andreas and Brunborg (30)	Norway	longitudinal	1,621	915		PHQ-9	Grades 8–10			8.17 ± 8.45	8.58 ± 7.41
Bélanger et al. (29)	Canada	longitudinal	7,160	1,863	GAD-7		Grades 9–12	6.06 ± 5.51	6.23 ± 5.44		
Black et al. (26)	United States	Cross-sectional	362	362		MFQ	8–17			10.28 ± 5.57	12.02 ± 5.13
Westrupp et al. (33)	Australia	Cross-sectional	2,365	2,365		MFQ	11–12			4.28 ± 4.58	5.04 ± 12.35
Mayne et al. (32)	United States	Cross-sectional	4,3504	4,7684		PHQ-9	15.3 (SD: 2.1)			8,937 (20.54)	10,929 (22.92)
Gladstone et al. (31)	United States	longitudinal	228	228		PHQ-8	12–18			3.44 ± 4.55	4.37 ± 4.88
Jolliff et al. (28)	United States	Cross-sectional	100	134	GAD-7	PHQ-8	13–17	4.67 ± 5.22	5.37 ± 5.18	5.27 ± 5.06	6.81 ± 5.67

a Pre COVID-19.

b Post COVID-19.

c SHSS: senior high school students.

### Meta-analysis results of anxiety

#### Combined effect

**Figure 2:**
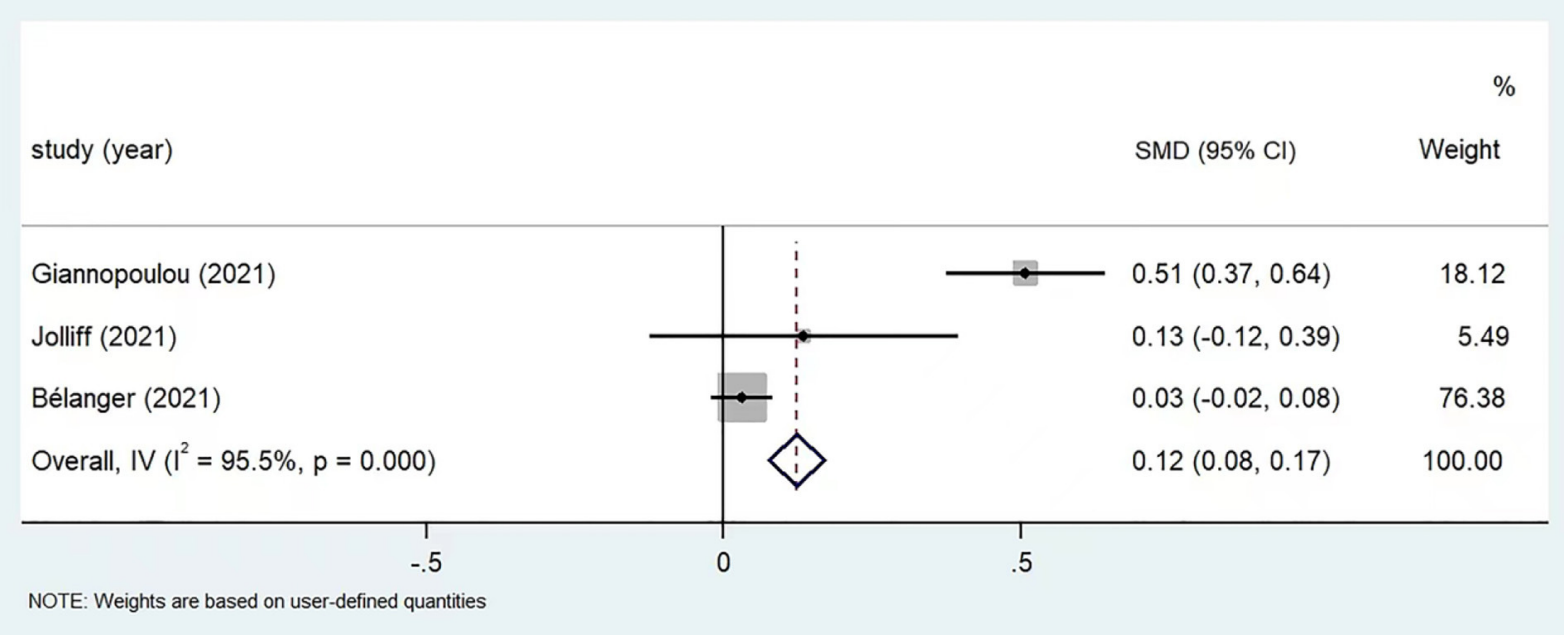
Meta-analysis of anxiety score before and post COVID-19 in children and adolescents (*n* = 3).

A meta-analysis was performed on the 3 included articles that measured anxiety by using the GAD-7. The means together with their standard deviations for GAD-7 scores pre and post the COVID-19 pandemic were compared, as shown in Figure 2. Among the three studies, Giannopoulou et al. (27) reported a significantly increased anxiety level in post COVID-19 period, with an SMD of 0.51 (95% CI: 0.37, 0.64), another two studies revealed insignificant differences between pre and post COVID-19 anxiety in children and adolescents, with SMDs of 0.13 (95% CI: −0.12, 0.39) and 0.03 (95% CI: −0.02, 0.08). Heterogeneity was significant among the three studies (*I*^2^= 95.5%, *p* < 0.05), therefore random-effects model was adopted. The combined SMD value was 0.12 (95% CI: 0.08, 0.17), suggesting a statistically higher anxiety level after COVID-19 pandemic.

#### Sensitivity analysis

As only 3 eligible studies were included for meta-analysis, regular statistical methods in detecting publication bias, such as Funnel plot and Begg's test, could not be applied. Therefore, we further performed a sensitivity analysis to evaluate the influence of individual studies. The results were presented in Supplementary Figure S1: among the three included studies, the exclusion of Giannopoulou et al. (27) or Bélanger et al. (29) prominently swayed the combined effect, whereas the study by Jolliff et al. (28) only showed negligible influence.

### Meta-analysis results of depression

#### Combined effect

**Figure 3:**
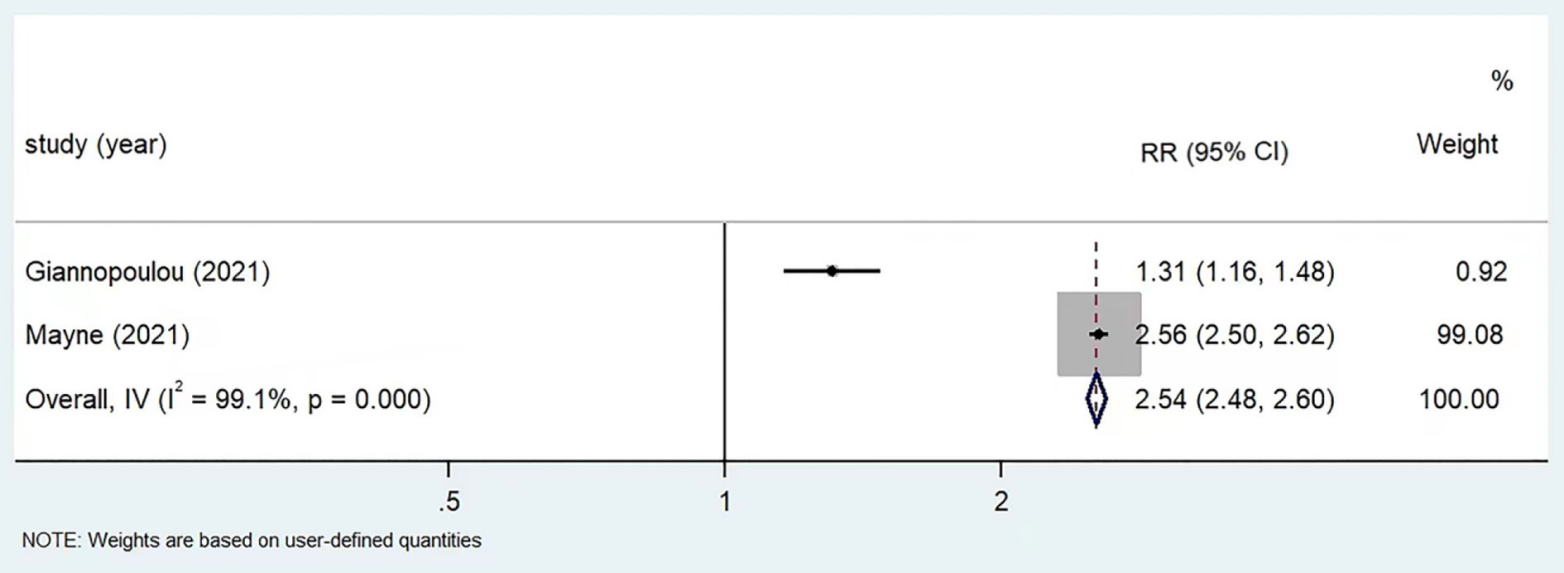
Meta-analysis of prevalence of depression before and post COVID-19 in children and adolescents by using PHQ-9 (*n* = 3).

A total of 7 studies provided data on depression in children and adolescents before and after the COVID-19 pandemic: 5 studies reported depression scores based on different instruments, 1 study reported both score and prevalence of depression, 1 study only reported prevalence of depression. Considering heterogeneity in instruments and outcome measurement, meta-analysis was performed for different subgroups of studies separately. For the two studies reported prevalence rates of depression, a statistically higher depression rate had been revealed, with RR values of 1.31 (95% CI: 1.16, 1.48) for Giannopoulou et al. (27), 2.56 (95% CI: 2.50, 2.62) for Mayne et al. (32). The combined RR was 2.54 (95% CI: 2.48, 2.60) based on random-effect model (*I*^2^= 99.1%, *p* < 0.05) (Figure 3).

**Figure 4:**
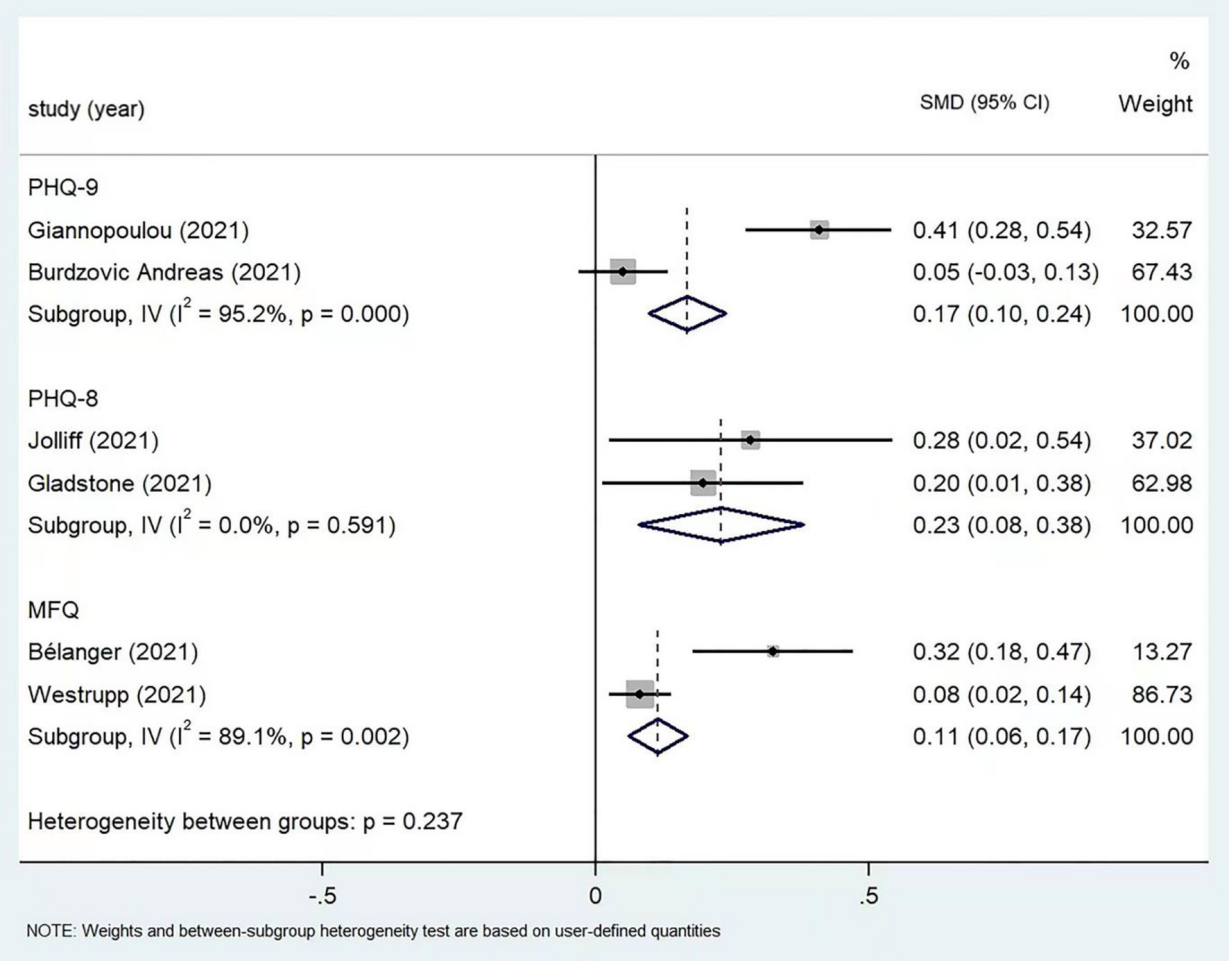
Subgroup meta-analysis of depression scores by using different scales (*n* = 6).

Mean and standard deviation for depression scores were reported in 6 articles using PHQ-9, PHQ-8, and MFQ, respectively. The results for subgroup analysis by different measuring scales were collectively shown in Figure 4. For the two studies used PHQ-9, Giannopoulou et al. (27) reported statistically increased depression score in post COVID-19 period, with an SMD of 0.41 (95% CI: 0.28, 0.54), whereas for Andreas and Brunborg (30), the increase was insignificant (SMD: 0.05, 95% CI: −0.03, 0.13). The two studies used PHQ-8 all found higher depression score after COVID-19 pandemic, with SMDs of 0.28 (95% CI: 0.02, 0.54) for Jolliff et al. (28) and 0.20 (95% CI: 0.01, 0.38) for Gladstone et al. (31). The two studies used MFQ also yielded positive findings, with SMDs of 0.32 (95% CI: 0.18, 0.47) for Black et al. (26) and 0.08 (95% CI: 0.02, 0.14) for Westrupp et al. (33). Meta-analysis results based on random-effect model revealed statistically increased depression score in post COVID-19 period for all three subgroups: the combined SMDs were 0.17 (95% CI: 0.10, 0.24), 0.23 (95% CI: 0.08, 0.38), and 0.11 (95% CI: 0.06, 0.17) for PHQ-9, PHQ-8, and MFQ, respectively.

#### Sensitivity analysis

As for each depression instrument, only two eligible studies were included, sensitivity analysis for depression would be unnecessary.

## Discussion

Although a plenty of studies had been published in the past several years on the negative psychological impact of the COVID-19 in children and adolescents, only a few of them compared the difference in depression or anxiety levels pre and post the pandemic within the same or comparable populations, and with incongruent conclusions. The need to synthesize existing evidence on this topic is urgent, in the purpose of generating stronger evidence for intervention. In this meta-analysis, we systematically searched, screened, evaluated, and combined published studies of higher quality, and found that for children and adolescents, their post COVID-19 anxiety and depression levels were significantly higher than pre-pandemic period. This major finding suggests the necessity of targeted intervention measures for this psychologically vulnerable population in post-pandemic era.

The COVID-19 pandemic has brought huge changes in lifestyle of children and adolescents, and these changes may act as environmental stressors for mood fluctuation. For instance, considering that novel coronavirus transmission is mainly through respiratory droplets and close contact, young people may actively avoid social activities in the fear of contracting the virus. Besides, due to the COVID-19 lockdown, children and adolescents in many areas were confined to their homes for a long time. It has been wide recognized that social isolation is associated with higher risk of depression and anxiety in children and adolescents (42, 43). In addition, children and adolescents spend less time in physical activity than they did before the COVID-19 pandemic, and some studies have shown that children and adolescents who lack of physical exercise were at significantly increased risk of anxiety and depression (44). As children and young people facing the continuing coronavirus pandemic, the fear that they or their friends or family members will be infected by the virus had been shown associated with an increase in anxiety and depressive symptoms during COVID-19 pandemic (45). It has also been found that the global economic recession caused by COVID-19 pandemic had an impact on depression and anxiety in children and adolescents, especially for individuals from families with low socioeconomic status (46–48). Moreover, many studies have shown that COVID-19 also has a negative impact on parents' mental health (49, 50), and it has been reported that anxiety and depression of parents can be transmitted to children (51). Finally, as children and adolescents are isolated at home for longer periods of time and parents' pressure is increased due to isolation, the risk of child abuse in isolation period may also increase (52). It has been proved that maltreatment is associated with elevated risk of anxiety and depression in children and adolescents (53, 54).

Some important mechanisms might be involved in explaining deteriorated anxiety and depression status for children and adolescents in post COVID-19 period. At first, numerous environmental stressors generated by the pandemic can trigger neuroinflammation, a process intimately associated with dysfunctional hypothalamic-pituitary-adrenal (HPA) axis, that can lead to increased risk of mood disorders such as depression and anxiety (55, 56). Besides, longitudinal evidence suggests that adolescents under significant traumatic stress exhibited developmental increases in amygdala reactivity (57), and amygdala hyperactivity in adolescence had been associated with affect dysregulation (58). Moreover, COVID-19 may also cause brain structure and function changes that can contribute to aggravated psychiatric conditions. For instance, in a recently published multimodal magnetic resonance imaging study, the authors observed that widespread lower diffusivity among the main axis of white matter (WM), and abnormal functional connectivity (FC) among resting state networks were prominently associated with worse self-rated depression in 42 COVID-19 survivors (59).

The mental health conditions triggered by the COVID-19 pandemic, if left untreated, can compromise mental or even physical health of children and adolescents, and the adverse effect can extend into adulthood (60, 61). Therefore, effective and feasible intervention measures should be taken to prevent increased risk of anxiety and depression in children and adolescents. In COVID-19 pandemic background, expedient methods that compatible with infection containment policy should be devised and given priority. Some scholars have demonstrated that internet-based integrated interventions were effective for moderate anxiety and depression (62). An Italian study pointed out that in response to the psycho-social impact of the COVID-19 pandemic on adolescents, schools should organize online courses and social platforms not just for educational purpose, but also for adolescents to function socially (63). A newly published study revealed that a traditional Chinese physical exercise (Qigong) based intervention in combination with psychological support form of peer education can significantly reduce anxiety and depression in adolescents, which can help develop a mental health maintenance plan for adolescents living and studying at home during the COVID-19 pandemic (64).

This is the first comparative meta-analysis on depression and anxiety of children and adolescents pre and post COVID-19. Major findings of this study can provide important evidence for depression and anxiety intervention in children and adolescents during the COVID-19 pandemic. However, the current study has some limitations. First, as only a small number of eligible studies had been found and synthesized, our major findings should be further corroborated by future longitudinal studies of large samples and different origins. Second, for some of the included studies, pre COVID-19 depression and anxiety were measured by recall, therefore the possibility of information bias exists. Third, considering that for nearly all included studies, the time interval between pre and post COVID-19 measurements surpassed 6 months, it is possible that the change in symptoms of depression or anxiety was not a consequence of the pandemic. Finally, conspicuous heterogeneity existed between included studies, as suggested by sensitivity analysis, therefore, cautions should be taken when interpreting the combined results.

## Conclusion

In this meta-analysis, by synthesizing timely evidence, we found that for children and adolescents, their anxiety and depression status significantly deteriorated in post COVID-19 period. This major finding suggests unneglectable psychological impact of the COVID-19 on youths. More attention should be paid to this vulnerable population during the pandemic. Effective, expedient and practical intervention measures which are compatible with COVID-19 prevention and control policies should be designed, evaluated, and implemented to maintain mental health wellbeing of children and adolescents in post COVID-19 era.

## Data availability statement

The original contributions presented in the study are included in the article/Supplementary material, further inquiries can be directed to the corresponding author.

## Author contributions

YX conceived and designed this study. HR and YC were responsible for literature search and study selection. DF and XL were responsible for quality assessment. JP and HS were responsible for data extraction. SW and LC performed analyses and wrote the original manuscript. YX critically reviewed and revised the manuscript. All authors contributed to the final version of the manuscript. All authors contributed to the article and approved the submitted version.

## Funding

This study was funded by National Natural Science Foundation of China (82060601), Top Young Talents of Yunnan Ten Thousand Talents Plan (YNWR-QNBJ-2018-286), Innovative Research Team of Yunnan Province (202005AE160002).

## Conflict of interest

The authors declare that the research was conducted in the absence of any commercial or financial relationships that could be construed as a potential conflict of interest.

## Publisher's note

All claims expressed in this article are solely those of the authors and do not necessarily represent those of their affiliated organizations, or those of the publisher, the editors and the reviewers. Any product that may be evaluated in this article, or claim that may be made by its manufacturer, is not guaranteed or endorsed by the publisher.

## References

[1] Ferretti L Wymant C Kendall M Zhao L Nurtay A Abeler-Dörner L. Quantifying SARS-CoV-2 transmission suggests pandemic control with digital contact tracing. Science. (2020) 368:eabb6936. 10.1126/science.abb6936 32234805 PMC7164555

[2] Abbott A. COVID's mental-health toll: how scientists are tracking a surge in depression. Nature. (2021) 590:194–5. 10.1038/d41586-021-00175-z 33536600

[3] Cheng SKW Wong CW Tsang J Wong KC. Psychological distress and negative appraisals in survivors of severe acute respiratory syndrome (SARS). Psychol Med. (2004) 34:1187–95. 10.1017/S0033291704002272 15697045

[4] Park KH Kim AR Yang MA Lim SJ Park JH. Impact of the COVID-19 pandemic on the lifestyle, mental health, and quality of life of adults in South Korea. PLoS ONE. (2021) 16:e0247970. 10.1371/journal.pone.0247970 33635897 PMC7909697

[5] Xiang YT Yang Y Li W Zhang L Zhang Q Cheung T. Timely mental health care for the 2019 novel coronavirus outbreak is urgently needed. Lancet Psychiatry. (2020) 7:228–9. 10.1016/S2215-0366(20)30046-8 32032543 PMC7128153

[6] Kang LJ Li Y Lu SH Chen M Yang C Yang BX. The mental health of medical workers in Wuhan, China dealing with the 2019 novel coronavirus. Lancet Psychiatry. (2020) 7:E14. 10.1016/S2215-0366(20)30047-X 32035030 PMC7129673

[7] Ju YM Zhang Y Wang XP Li WH Ng RMK Li J. China's mental health support in response to COVID-19: progression, challenges and reflection. Global Health. (2020) 16:102. 10.1186/s12992-020-00634-8 33092606 PMC7578578

[8] Vindegaard N Benros ME. COVID-19 pandemic and mental health consequences: systematic review of the current evidence. Brain Behav Immun. (2020) 89:531–42. 10.1016/j.bbi.2020.05.048 32485289 PMC7260522

[9] Statistic Canada,. Canadian's Mental Health During the COVID-19 Pandemic. (2020). Available online at: https://www150.statcan.gc.ca/n1/daily-quotidien/200527/dq200527b-eng.htm (accessed November 6, 2021).

[10] Centre for Addictions Mental Health. COVID-19 National Survey Dashboard. (2020). Available online at: https://www.camh.ca/en/health-info/mental-health-and-covid-19/covid-19-national-survey (accessed November 6, 2021).

[11] Turna J Zhang J Lamberti N Patterson B Simpson W Francisco AP. Anxiety, depression and stress during the COVID-19 pandemic: results from a cross-sectional survey. J Psychiatr Res. (2021) 137:96–103. 10.1016/j.jpsychires.2021.02.059 33667763 PMC8548032

[12] Mautong H Gallardo-Rumbea JA Alvarado-Villa GE Fernández-Cadena JC Andrade-Molina D Orellana-Román CE. Assessment of depression, anxiety and stress levels in the Ecuadorian general population during social isolation due to the COVID-19 outbreak: a cross-sectional study. BMC Psychiatry. (2021) 21:212. 10.1186/s12888-021-03214-1 33910550 PMC8080088

[13] Chen X. The Relationship of college students'negative mood regulation expectancies, negative life events and state anxiety. China J Health Psychol. (2015) 23:295–9. 10.13342/j.cnki.cjhp.2015.02.037

[14] Xie XY Xue Q Zhou Y Zhu KH Liu Q Zhang JJ. Mental health status among children in home confinement during the coronavirus disease 2019 outbreak in Hubei Province, China. JAMA Pediatr. (2020) 174:898–900. 10.1001/jamapediatrics.2020.1619 32329784 PMC7182958

[15] Zhou SJ Zhang LG Wang LL Guo ZC Wang JQ Chen JC. Prevalence and socio-demographic correlates of psychological health problems in Chinese adolescents during the outbreak of COVID-19. Eur Child Adolesc Psychiatry. (2020) 29:749–58. 10.1007/s00787-020-01541-4 32363492 PMC7196181

[16] Fong VC Larocci G. Child and family outcomes following pandemics: A systematic review and recommendations on COVID-19 policies. J Pediatr Psychol. (2020) 45:1124–43. jsaa092. 10.1093/jpepsy/jsaa092 33083817 PMC7665615

[17] Samji H We J Ladak A Vossen C Stewart E Dove N. Review: Mental health impacts of the COVID-19 pandemic on children and youth-a systematic review. Child Adolescent Mental Health. (2022) 27:173–89. 10.1111/camh.12501 34455683 PMC8653204

[18] Racine N McArthur BA Cook JE Eirich R Zhu J Madigan S. Global prevalence of depressive and anxiety symptoms in children and adolescents during covid-19: a meta-analysis. JAMA Pediatr. (2021) 175:1142–50. 10.1001/jamapediatrics.2021.2482 34369987 PMC8353576

[19] Li Y Zhou Y Ru TT Niu JX He MH Zhou GF. How does the COVID-19 affect mental health and sleep among Chinese adolescents: a longitudinal follow-up study. Sleep Med. (2021) 85:246–58. 10.1016/j.sleep.2021.07.008 34388503 PMC8418314

[20] Hollenstein T Colasante T Lougheed JP. Adolescent and maternal anxiety symptoms decreased but depressive symptoms increased before to during COVID-19 lockdown. J Res Adolesc. (2021) 31:517–30. 10.1111/jora.12663 34448298 PMC8646576

[21] Moher D Liberati A Tetzlaff J Altman DG. Preferred reporting items for systematic reviews and meta-analyses: the PRISMA statement. Ann Intern Med. (2009) 151:264–W64. 10.7326/0003-4819-151-4-200908180-00135 19622511

[22] The Ottawa Hospital Research Institute. NOS. (2015). Available online at: http://www.ohri.ca/programs/clinical_epidemiology/oxford.asp (accessed December 14, 2015).

[23] Founou RC Founou LL Essack SY. Clinical and economic impact of antibiotic resistance in developing countries: a systematic review and meta-analysis. PLoS ONE. (2017) 12:e0189621. 10.1371/journal.pone.0189621 29267306 PMC5739407

[24] Ma L Mazidi M Li K Li YX Chen SQ Kirwan R. Prevalence of mental health problems among children and adolescents during the COVID-19 pandemic: a systematic review and meta-analysis. J Affect Disord. (2021) 293:78–89. 10.1016/j.jad.2021.06.021 34174475 PMC9711885

[25] Bronsard G Alessandrini M Fond G Loundou A Auquier P Tordjman S. The prevalence of mental disorders among children and adolescents in the child welfare system: a systematic review and meta-analysis. Medicine. (2016) 95:e2622. 10.1097/MD.0000000000002622 26886603 PMC4998603

[26] Black SR Evans ML Aaron L Brabham DR Kaplan RM. Covariance between parent and child symptoms before and during the COVID-19 pandemic. J Pediatr Psychol. (2021) 46:1182–94. 10.1093/jpepsy/jsab086 34405876 PMC8385985

[27] Giannopoulou I Efstathiou V Triantafyllou G Korkoliakou P Douzenis A. Adding stress to the stressed: Senior high school students' mental health amidst the COVID-19 nationwide lockdown in Greece. Psychiatry Res. (2021) 295:113560. 10.1016/j.psychres.2020.113560 33187723 PMC7647459

[28] Jolliff A Zhao Q Eickhoff J Moreno M. Depression, anxiety, and daily activity among adolescents before and during the COVID-19 pandemic: cross-sectional survey study. JMIR Form Res. (2021) 5:e30702. 10.2196/30702 34609316 PMC8641699

[29] Bélanger RE Patte KA Leatherdale ST Gansaonré RJ Haddad S. An impact analysis of the early months of the COVID-19 pandemic on mental health in a prospective cohort of Canadian adolescents. J Adolesc Health. (2021) 69:917–24. 10.1016/j.jadohealth.2021.07.039 34565667 PMC8457891

[30] Andreas JB Brunborg GS. Self-reported mental and physical health among Norwegian adolescents before and during the COVID-19 pandemic. JAMA Netw Open. (2021) 4:e2121934–e2121934. 10.1001/jamanetworkopen.2021.21934 34427678 PMC8385591

[31] Gladstone TR Schwartz JA Pössel P Richer AM Buchholz KR Rintell L. Depressive symptoms among adolescents: Testing vulnerability-stress and protective models in the context of COVID-19. Child Psychiatry Hum Dev. (2021) 7:1–11. 10.1007/s10578-021-01216-4 34235630 PMC8262586

[32] Mayne SL Hannan C Davis M Young JF Kelly MK Powell M. COVID-19 and adolescent depression and suicide risk screening outcomes. Pediatrics. (2021) 148:e2021051507. 10.1542/peds.2021-051507 34140393

[33] Westrupp EM Bennett C Berkowitz T Youssef GJ Toumbourou JW Tucker R. Child, parent, and family mental health and functioning in Australia during COVID-19: Comparison to pre-pandemic data. Eur Child Adolesc Psychiatry. (2021) 21:1–14. 10.1007/s00787-021-01861-z 34417875 PMC8379590

[34] Leung DYP Mak YW Leung SF Chiang VCL Loke AY. Measurement invariances of the PHQ-9 across gender and age groups in Chinese adolescents. Asia-Pac Psychiatry. (2020) 12:e12381. 10.1111/appy.12381 32011101 PMC7507123

[35] Wu Y Levis B Riehm KE Saadat N Levis AW Azar M. Equivalency of the diagnostic accuracy of the PHQ-8 and PHQ-9: a systematic review and individual participant data meta-analysis. Psychol Med. (2020) 50:2816–2816. 10.1017/S0033291719002137 31423953

[36] Kroenke K Spitzer RL Williams JBW. The PHQ-9: validity of a brief depression severity measure. J Gen Intern Med. (2001) 16:606–13. 10.1046/j.1525-1497.2001.016009606.x 11556941 PMC1495268

[37] Shin C Lee SH Han KM Yoon HK Han C. Comparison of the usefulness of the PHQ-8 and PHQ-9 for screening for major depressive disorder: analysis of psychiatric outpatient data. Psychiatry Investig. (2019) 16:300–5. 10.30773/pi.2019.02.01 31042692 PMC6504773

[38] Wood A Kroll L Moore A Harrington R. Properties of the mood and feelings questionnaire in adolescent psychiatric outpatients: a research note. J Child Psychol Psychiatry. (1995) 36:327–34. 10.1111/j.1469-7610.1995.tb01828.x 7759594

[39] Burleson Daviss W Birmaher B Melhem NA Axelson DA Michaels SM Brent DA. Criterion validity of the Mood and Feelings Questionnaire for depressive episodes in clinic and non-clinic subjects. J Child Psychol Psychiatry. (2006) 47:927–34. 10.1111/j.1469-7610.2006.01646.x 16930387

[40] NHIS−2019 NHIS. (2021). Available online at: https://www.cdc.gov/nchs/nhis/2019nhis.htm (accessed June 5, 2021).

[41] Tiirikainen K Haravuori H Rant K Kaltiala-Heino R Marttunen M. Psychometric properties of the 7-item Generalized Anxiety Disorder Scale (GAD-7) in a large representative sample of Finnish adolescents. Psychiatry Res. (2019) 272:30–5. 10.1016/j.psychres.2018.12.004 30579178

[42] Song MK Lee JH Kim YJ. Effect of chronic handling and social isolation on emotion and cognition in adolescent rats. Physiol Behav. (2021) 237:113440. 10.1016/j.physbeh.2021.113440 33940083

[43] Loades ME Chatburn E Higson-Sweeney N Reynolds S Shafran R Brigden A. Rapid systematic review: the impact of social isolation and loneliness on the mental health of children and adolescents in the context of COVID-19. J Am Acad Child Adolesc Psychiatry. (2020) 59:1218–39. e3. 10.1016/j.jaac.2020.05.009 32504808 PMC7267797

[44] Carter T Pascoe M Bastounis A Morres ID Callaghan P Parker AG. The effect of physical activity on anxiety in children and young people: A systematic review and meta-analysis. J Affect Disord. (2021) 285:10–21. 10.1016/j.jad.2021.02.026 33618056

[45] Deolmi M Pisani F. Psychological and psychiatric impact of COVID-19 pandemic among children and adolescents. Acta Biomed. (2020) 91:e2020149. 10.23750/abm.v91i4.10870 33525229 PMC7927507

[46] Lemstra M Neudorf C D'Arcy C Kunst A Warren LM Bennett NR. systematic review of depressed mood and anxiety by SES in youth aged 10–15 years. Can J Public Health. (2008) 99:125–9. 10.1007/BF03405459 18457287 PMC6975760

[47] Quon EC McGrath JJ. Community, family, and subjective socioeconomic status: Relative status and adolescent health. Health Psychol. (2015) 34:591–601. 10.1037/hea0000135 25222085 PMC5756086

[48] Xiaochen Y Jingfen Z Ziyue C Yinliang T Yaping HE. Influence of individual and family characteristics on middle school students' mental health status during COVID-19 pandemic. Scl Health China. (2021) 42:858–62. 10.16835/j.cnki.1000-9817.2021.06.014

[49] Wu M Xu W Yao Y Zhang L Guo L Fan J. Mental health status of students' parents during COVID-19 pandemic and its influence factors. Gen Psychiatry. (2020) 33:e100250. 10.1136/gpsych-2020-100250 34192232 PMC7387315

[50] Calvano C Engelke L Di Bella J Kindermann J Renneberg B Winter SM. Families in the COVID-19 pandemic: parental stress, parent mental health and the occurrence of adverse childhood experiences—results of a representative survey in Germany. Eur Child Adolesc Psychiatry. (2021) 1:1–13. 10.1007/s00787-021-01739-0 33646416 PMC7917379

[51] Aktar E Nikoli M Bgels SM. Environmental transmission of generalized anxiety disorder from parents to children: Worries, experiential avoidance, and intolerance of uncertainty. Dialogues Clin Neurosci. (2017) 19:137–47. 10.31887/DCNS.2017.19.2/eaktar 28867938 PMC5573558

[52] Rosenthal CM Thompson LA. Child abuse awareness month during the coronavirus disease 2019 pandemic. JAMA Pediatr. (2020) 174:812–812. 10.1001/jamapediatrics.2020.1459 32329789

[53] Harkness KL Lumley MN. Child abuse and neglect and the development of depression in children and adolescents. In: Abela JRZ, Hankin BL, editors. Handbook of Depression in Children and Adolescents. New York, NY: The Guilford Press (2008). p. 466–488.

[54] Leenarts LEW Diehle J Doreleijers TAH Jansma EP Lindauer RJL. Evidence-based treatments for children with trauma-related psychopathology as a result of childhood maltreatment: a systematic review. Eur Child Adolesc Psychiatry. (2013) 22:269–83. 10.1007/s00787-012-0367-5 23266844

[55] Jiang NM Cowan M Moonah SN Petri WA Jr. The Impact of Systemic Inflammation on Neurodevelopment. Trends Mol Med. (2018) 24:794–804. 10.1016/j.molmed.2018.06.008 30006148 PMC6110951

[56] Calcia MA Bonsall DR Bloomfield PS Selvaraj S Barichello T Howes OD. Stress and neuroinflammation: a systematic review of the effects of stress on microglia and the implications for mental illness. Psychopharmacology. (2016) 233:1637–50. 10.1007/s00213-016-4218-9 26847047 PMC4828495

[57] Tottenham N Galvan A. Stress and the adolescent brain: amygdala-prefrontal cortex circuitry and ventral striatum as developmental targets. Neurosci Biobehav Rev. (2016) 70:217–27. 10.1016/j.neubiorev.2016.07.030 27473936 PMC5074883

[58] Swartz JR Knodt AR Radtke SR Hariri AR. A neural biomarker of psychological vulnerability to future life stress. Neuron. (2015) 85:505–11. 10.1016/j.neuron.2014.12.055 25654256 PMC4319095

[59] Benedetti F Palladini M Paolini M Melloni E Vai B De Lorenzo R. Brain correlates of depression, post-traumatic distress, and inflammatory biomarkers in COVID-19 survivors: a multimodal magnetic resonance imaging study. Brain Behav Immun Health. (2021) 18:100387. 10.1016/j.bbih.2021.100387 34746876 PMC8562046

[60] Golberstein E Wen H Miller BF. Coronavirus Disease 2019 (COVID-19) and Mental Health for Children and Adolescents. JAMA Pediatr. (2020) 174:819–20. 10.1001/jamapediatrics.2020.1456 32286618

[61] Binagwaho A Senga J. Children and adolescent mental health in a time of COVID-19: a forgotten priority. Ann Glob Health. (2021) 87:57. 10.5334/aogh.3330 34249619 PMC8252973

[62] Wei N Huang BC Lu SJ Hu JB Zhou XY Hu CC. Efficacy of internet-based integrated intervention on depression and anxiety symptoms in patients with COVID-19. J Zhejiang Univ Sci B. (2020) 21:400–4. 10.1631/jzus.B2010013 32425006 PMC7203540

[63] Buzzi C Tucci M Ciprandi R Brambilla I Caimmi S Ciprandi G. The psycho-social effects of COVID-19 on Italian adolescents' attitudes and behaviors. Ital J Pediatr. (2020) 46:69. 10.1186/s13052-020-00833-4 32448323 PMC7245982

[64] Ding X Yao J. Peer Education Intervention on Adolescents' Anxiety, Depression, and Sleep Disorder during the COVID-19 Pandemic. Psychiatr Danub. (2020) 32:527–35. 10.24869/psyd.2020.527 33370763

