# Supplementary material for: Depression and anxiety among children and adolescents pre and post COVID-19: A comparative meta-analysis
Source: Front Psychiatry. 2022 Aug 3;13:917552. doi: 10.3389/fpsyt.2022.917552 (PMC9381924; doi:10.3389/fpsyt.2022.917552)
Supplement: Supplementary file 1 [file Data_Sheet_1.docx]

Table S1 The NOS rating scores for included studies.

| First author/Publication year | Scores | | |
| --- | --- | --- | --- |
|  | Selection | Comparability | Outcome |
| Giannopoulou/2021 | 3 | 1 | 3 |
| Burdzovic Andreas/2021 | 2 | 1 | 3 |
| Bélanger/2021 | 3 | 2 | 2 |
| Black/2021 | 2 | 2 | 2 |
| Westrupp/2021 | 2 | 1 | 2 |
| Mayne/2021 | 2 | 2 | 3 |
| Gladstone/2021 | 2 | 1 | 2 |
| Jolliff/2021 | 3 | 1 | 3 |


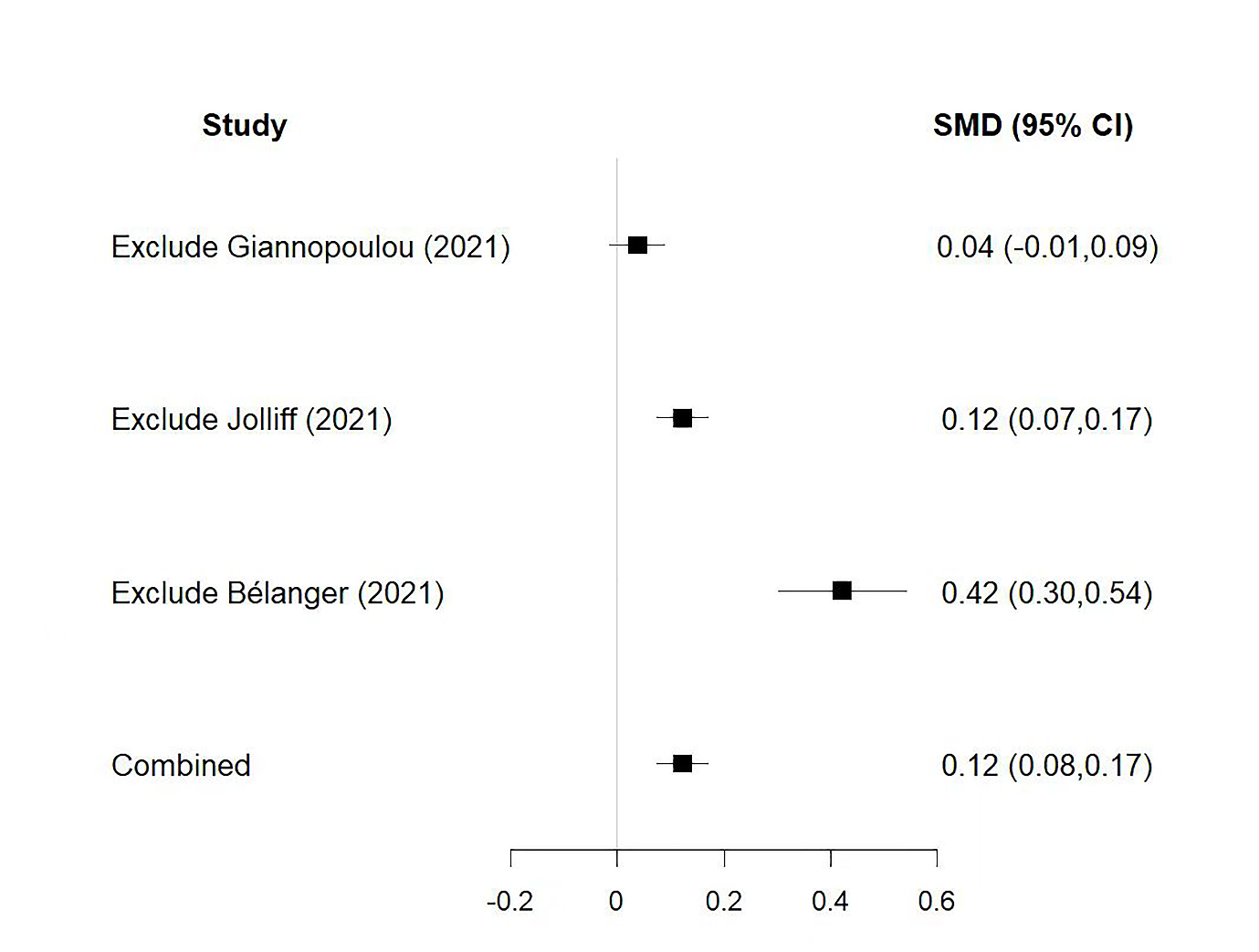


Figure S1 Sensitivity analysis for anxiety scores SMD pre and post COVID-19 in children and adolescents
